# Acute patient‐reported outcomes in B‐cell malignancies treated with axicabtagene ciloleucel

**DOI:** 10.1002/cam4.3664

**Published:** 2021-02-28

**Authors:** Aasha I. Hoogland, Reena V. Jayani, Aaron Collier, Nathaly Irizarry‐Arroyo, Yvelise Rodriguez, Michael D. Jain, Margaret Booth‐Jones, Kelly A. Hyland, Brian W. James, Anna Barata, Christina A. Bachmeier, Julio C. Chavez, Farhad Khimani, Gabriel S. Krivenko, Aleksandr Lazaryan, Hien D. Liu, Taiga Nishihori, Javier Pinilla‐Ibarz, Bijal D. Shah, Muneer Abidi, Frederick L. Locke, Heather S. L. Jim

**Affiliations:** ^1^ Moffitt Cancer Center Department of Health Outcomes and Behavior Tampa FL USA; ^2^ Vanderbilt University Medical Center Department of Medicine Division of Hematology/Oncology Nashville TN USA; ^3^ Moffitt Cancer Center Department of Blood and Marrow Transplant and Cellular Immunotherapy Tampa FL USA; ^4^ Moffitt Cancer Center Department of Supportive Care Medicine Tampa FL USA; ^5^ Moffitt Cancer Center Department of Malignant Hematology Tampa FL USA; ^6^ Spectrum Health Cancer Center Michigan State University Grand Rapids MI USA

**Keywords:** behavioral science, hematological cancer, lymphoma, quality of life

## Abstract

Chimeric antigen receptor T‐cell therapy with axicabtagene ciloleucel (axi‐cel) has considerably improved survival in adults with relapsed/refractory large B‐cell lymphoma. This study reports patient‐reported outcomes (PROs) such as quality of life (QOL) and toxicity in the first 90 days after treatment. Hematologic cancer patients treated with axi‐cel (*N* = 103, mean age = 61, 39% female) completed SF‐36 or PROMIS‐29 QOL questionnaires prior to treatment and 90 days after. PRO‐Common Terminology Criteria for Adverse Events toxicity items were completed by patients at baseline and 14, 30, 60, and 90 days after treatment. Mixed models examined change in PROs over time. From preinfusion to 90 days later, patients reported improvements in physical functioning, pain, and fatigue (*p*s < 0.01), but worsening of anxiety (*p* = 0.02). Patient‐reported toxicities worsened by day 14 with improvement thereafter. The five most severe symptoms at day 14 included fatigue, decreased appetite, dry mouth, diarrhea frequency, and problems with concentration. Results indicate improvement in some domains of QOL over time with transient patient‐reported toxicities.

## Graphical abstract

From pre‐chimeric antigen receptor (CAR) T‐cell therapy infusion to 90 days later, there were significant improvements in physical functioning and reductions in pain and fatigue, but a significant worsening of anxiety. Patient‐reported toxicities worsened by day 14 with improvement thereafter.

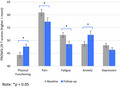

## 1 INTRODUCTION

The chimeric antigen receptor (CAR) T‐cell therapy axicabtagene ciloleucel (axi‐cel) has generated widespread excitement for its ability to induce complete response in adult patients with relapsed/refractory large B‐cell lymphoma (LBCL).^1^, ^2^ A Phase I/II study of axi‐cel reported durable responses beyond 2 years in 39% of patients after a single infusion.^1^, ^2^ The median overall survival (OS) was not reached at 27.7 months follow‐up.^1^ These outcomes compare favorably to historical data showing refractory LBCL patients have a 26% chance for any response, 7% chance for complete response, and a median OS of less than 6 months with traditional chemotherapy.^3^ As such, additional studies of axi‐cel are currently underway in a variety of hematologic malignancies such as other non‐Hodgkin's lymphomas (NHL) and relapsed/refractory acute lymphoblastic leukemia (ALL).

Chimeric antigen receptor T‐cell therapy is characterized by potentially life‐threatening side effects, including cytokine release syndrome (CRS) and neurologic toxicity.^4^ CRS is caused by inflammatory cytokines released by CAR T‐cells or other immune cells.^4^ Experienced by over 90% of axi‐cel recipients, the hallmark of CRS is fever which can be accompanied by mild constitutional symptoms, such as headache and malaise, to severe multiple organ system involvement, such as hypotension, arrhythmia, hypoxia, respiratory failure, and renal failure.^4^, ^5^ Less is understood about the pathophysiology of neurologic toxicity, which affects over two‐thirds of axi‐cel recipients.^1^, ^2^ One‐third of recipients have severe neurologic toxicity which can include severe expressive aphasia, obtundation, seizures, or cerebral edema.^1^, ^5^, ^6^, ^7^ These toxicities have been reported through standardized provider‐reported methods for toxicity grading, including Common Terminology Criteria for Adverse Events (CTCAE) grading and NCI Consensus criteria for CRS^6^; there are no published papers to our knowledge that describe patient‐reported outcomes (PROs) such as quality of life (QOL) or patient‐reported toxicities.

There is increasing awareness of the importance of collecting PRO data to understand the impact of cancer and its treatment,^8^, ^9^ including CAR T‐cell therapy. PROs are especially relevant to CAR T‐cell therapy given its unique toxicity profile and the logistics required to administer treatment and supportive care.^10^ PROs demonstrate significant associations with important clinical outcomes such as survival and performance status.^11^, ^12^, ^13^ PROs can also contribute to a more complete understanding of adverse events, as data suggest that concordance between clinicians' and patients' reports of symptoms is low.^14^, ^15^, ^16^ Regulatory agencies have placed increasing importance on PROs,^17^, ^18^ marking a major shift in recognition of PROs as complementary to other clinical data regarding novel oncology therapies. To our knowledge, only three published studies have reported on PROs in adult patients treated with CAR T‐cell therapy. Ruark et al^19^ described patient‐reported symptoms 1–5 years posttreatment among 40 patients with relapsed/refractory ALL, chronic lymphocytic leukemia, or NHL on a Phase I/II trial of CD‐19‐targeted CAR T‐cells. They reported that approximately half of patients reported clinically significant cognitive impairment, depression, or anxiety. Conversely, two Phase II trials of tisagenlecleucel suggest that in adult patients with relapsed/refractory DLBCL^20^ and pediatric and young adult patients with relapsed/refractory B‐cell ALL,^21^ QOL increases in the first 90 days after infusion. These data suggest that further studies are warranted, particularly during the acute treatment period.

The aims of the current observational study were to: (1) examine change in QOL from before to 90 days after axi‐cel is administered commercially or as part of a clinical trial, and (2) examine change in patient‐reported toxicities in the first 90 days after axi‐cel. CRS, neurologic toxicity, and response to therapy were explored for their potential associations with change in QOL. The goal of the study was to provide descriptive patient‐reported information to help patients and caregivers better understand what patients might experience during CAR T‐cell therapy.

## 2 METHODS

### 2.1 Participants

Patients receiving axi‐cel therapy were recruited prospectively between October 2016 and April 2019 as part of a larger observational study assessing PROs and neurocognitive outcomes in patients treated with CAR T‐cell therapy. Patients were eligible for the parent study if they were: (1) 18 years of age or older, (2) diagnosed with a hematologic malignancy, (3) scheduled to receive CAR T‐cell therapy at Moffitt Cancer Center commercially or as part of a clinical trial, (4) able to speak and read English, (5) free of documented or observable psychiatric or neurological diagnoses at study entry that could interfere with study participation, and (6) able to provide informed consent. The research protocol was approved by the Advarra Institutional Review Board and all participants provided written informed consent. The data that support the findings of this study are available from the corresponding author upon reasonable request.

### 2.2 Measures

#### 2.2.1 Demographic and clinical data

Demographic data were obtained prior to lymphodepleting chemotherapy and included age, gender, race, ethnicity, marital status, and education. Clinical data were collected via chart review and included disease type, number of prior lines of therapy, number of days hospitalized in the first 100 days, highest grades of CRS and neurologic toxicity, and response to axi‐cel therapy. Neurologic toxicity was initially graded by the CAR T‐cell‐related encephalopathy syndrome defined by the CAR T‐cell‐therapy‐associated TOXicity (CARTOX) Working Group.^5^ With an updated definition of neurologic toxicity by the American Society for Transplantation and Cellular Therapy, subsequent neurologic toxicity was graded based on Immune Effector Cell‐Associated Neurotoxicity Syndrome (ICANS).^6^ Both CARTOX and ICANS use a grading scale of 1–4, with higher grades indicating worse toxicity.

#### 2.2.2 Quality of life

Initially, QOL data were collected using the Medical Outcomes Study Short Form‐36 version (SF‐36).^22^ QOL was assessed before pre‐CAR lymphodepleting chemotherapy and 90 days after axi‐cel. The SF‐36 covers eight domains: vitality, bodily pain, physical function, role limitations due to physical health, role limitations due to emotional problems, emotional well‐being, social function, and general health. Domain scores can be summarized into physical and mental component summaries.

Following publication of the proposed Center for Medicare and Medicaid Services (CMS) coverage decision for CAR T‐cell therapy in February 2019,^17^ the study switched from the SF‐36 to the CMS‐recommended PROs Measurement Information System‐29 version 2.1 (PROMIS^®^‐29)^23^ to assess QOL. The study continued to use the PROMIS‐29 after release of the final CMS coverage decision in July 2019 which removed the mandate to capture PROs.^24^ The PROMIS^®^‐29 evaluates eight domains: physical function, anxiety, depression, fatigue, sleep disturbance, ability to participate in social roles and activities, pain interference, and pain intensity. The PROMIS^®^‐29 is increasingly being used in hematopoietic cell transplantation (HCT) recipients (who receive established cellular therapy for hematologic malignancies most similar to CAR T‐cell therapy),^25^, ^26^, ^27^ and previous studies have demonstrated PROMIS‐29 adequately captures HCT recipients' symptoms and well‐being.^28^ For the current analyses, SF‐36 scores were converted to PROMIS‐29 T‐scores using PROsetta Stone^®^.^29^ The PROsetta Stone^®^ enables data harmonization between PROMIS scores and those obtained by other measures assessing similar health outcomes.^30^ PROsetta Stone^®^ conversion yields the following outcomes: physical function, anxiety, depression, fatigue, and pain interference.^29^ Consequently, the current analyses focused on these outcomes. Higher scores indicate more of the attribute being measured. QOL items were keyed to the past week. QOL was not assessed on days 14, 30, and 60 to reduce participant burden.

#### 2.2.3 Patient‐reported toxicities

Patient‐reported toxicities were evaluated with the PROs Version of the CTCAE (PRO‐CTCAE).^31^ The PRO‐CTCAE allows for selection of relevant items from a 124‐item bank covering 78 symptomatic toxicities. Items assess the presence, frequency, severity, and/or interference with activities on a 5‐point Likert scale with higher scores indicating worse toxicity. The following items were selected: abdominal pain, constipation, cough, decreased appetite, diarrhea, dry mouth, fatigue, feeling sad, hair loss (yes/no), hand–foot syndrome, headache, insomnia, itchy skin, joint aches, muscle aches, nausea, problems with concentration, problems with memory, rash (yes/no), shortness of breath, and wheezing. These toxicities were selected based on symptomatic adverse events reported in the registration trial of axi‐cel^2^ as well as commonly reported adverse events of HCT and other immunotherapies. The PRO‐CTCAE was administered prior to the start of therapy and at 14, 30, 60, and 90 days posttreatment.

#### 2.2.4 Statistical analysis

Participants diagnosed with NHL who completed at least one PRO assessment were included in the current analyses. Means, standard deviations, confidence intervals, frequencies, and percentages were used to characterize participant characteristics and outcomes. Change in QOL was examined using mixed models, which allow for the use of all available data. Putative clinical moderators of change in QOL were explored using mixed models. Moderators included disease response (yes/no), cytokine release syndrome (low‐grade CRS = 0 or 1, high‐grade CRS = 2, 3, or 4), and neurologic toxicity (low‐grade ICANS = 0 or 1, high‐grade ICANS = 2, 3, or 4). Because self‐reported toxicities were evaluated in axi‐cel recipients at five time points, mixed models with linear and quadratic effects of time were used to evaluate change in toxicities controlling for baseline. All analyses were conducted in SAS, version 9.4 (Cary, NC) using an *α* of 0.05.

## 3 RESULTS

### 3.1 Descriptive characteristics

A CONSORT diagram is provided in Figure S1. Sociodemographic and clinical characteristics of participants are shown in Table 1. On average, axi‐cel recipients (*n* = 103) were 61 years of age, male, non‐Hispanic, White, and married. Approximately three‐quarters of participants demonstrated response to therapy.

**TABLE 1:** Participant characteristics.

	N = 103
Age: M (SD)	60.60 (12.27)
Sex: n (%) female	40 (39)
Ethnicity: n (%) non‐Hispanic	95 (93)
Race: n (%) white	90 (87)
Marital status: n (%) married	72 (71)
Education: n (%) college graduate	55 (54)
Inpatient days before day 100: M (SD)	15.92 (10.16)
Prior lines of therapy: n (%)	
1–3	65 (63)
≥4	38 (37)
Comorbidities: n (%)	
<3	63 (61)
3+	40 (39)
Disease response: n (%)	71 (74)
Max CRS: n (%)	
Grades 0 or 1	55 (53)
Grades 2–4	48 (47)
Max neurologic toxicity: n (%)	
Grades 0 or 1	74 (76)
Grades 2–4	24 (24)

### 3.2 Quality of life

**FIGURE 1:**
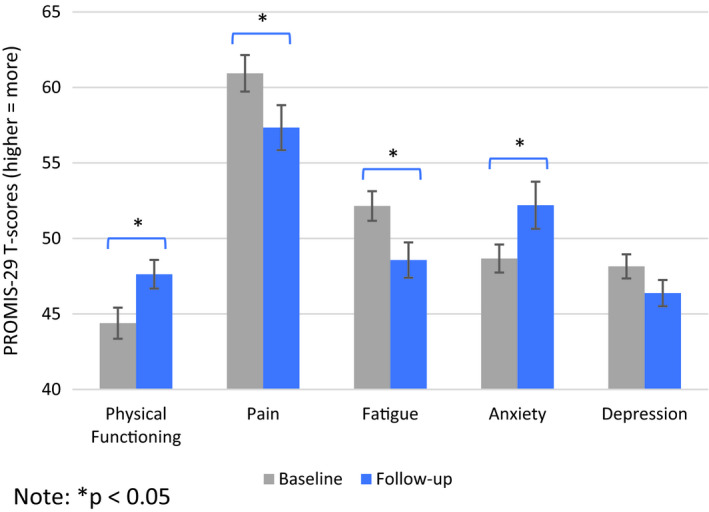
Quality of life over time, means, and SE bars.

Unadjusted means and SEs for each QOL outcome over time are presented in Figure 1. Over time, there were significant improvements in physical functioning and reductions in pain and fatigue (*p*s < 0.01). There was significant worsening of anxiety (*p* = .02). Changes in QOL were not associated with disease response (*p* > 0.59), CRS (*p* > 0.35), or neurologic toxicity (*p* > 0.25).

### 3.3 Toxicities

**FIGURE 2:**
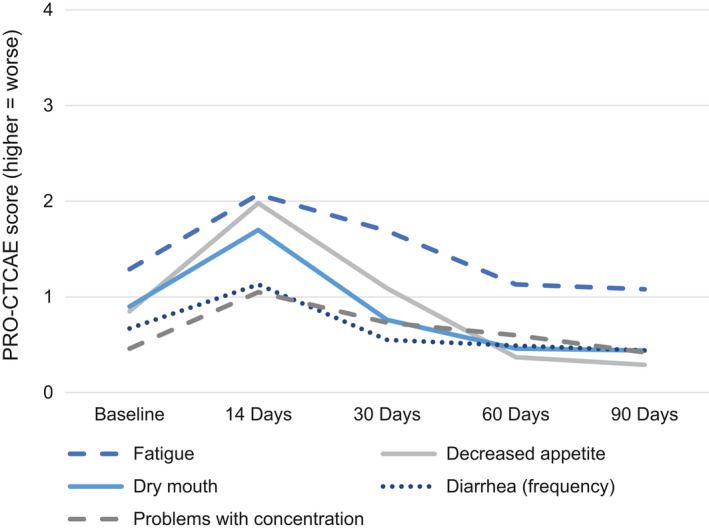
Trajectories of the five most severe patient‐reported toxicities at day 14 among patients treated with axi‐cel.

The highest severity of self‐reported toxicities at any time point are presented in Table 2. Means and standard deviations for toxicities are presented in Table S2; mixed models examining change over time in toxicities are presented in Table S3. There were significant peaks at the 14 day assessment followed by declines for dry mouth (*p* < 0.01), decreased appetite (*p* < 0.01), nausea (*p* < 0.01), cough (*p* = 0.02), hair loss (*p* < 0.01), hand–foot syndrome (*p* = 0.02), problems with concentration (*p* < 0.01), problems with memory (*p* < 0.01), headache (*p* < 0.01), and fatigue (*p* < 0.01). Aching muscles peaked at 14 days and remained stable over time (*p* < 0.05). Figure 2 depicts change over time in the top five most severe symptoms at 14 days as assessed using continuous scores (i.e., fatigue, decreased appetite, dry mouth, diarrhea frequency, and problems with concentration).

**TABLE 2:** Percentage of patients reporting toxicity at any time point.

Symptom	Any (%)	Moderate to very severe (%)
Fatigue	96	84
Decreased appetite	91	73
Dry mouth	89	61
Aching muscles	85	49
Insomnia	82	55
Sad or unhappy	80	34
Diarrhea (frequency)	76	46
Constipation	75	45
Problems with concentration	75	38
Headache	73	39
Shortness of breath	71	39
Aching joint	71	39
Problems with memory	69	32
Cough	64	29
Nausea	63	37
Abdominal pain	63	35
Hair loss (yes/no)	53	—
Itchy skin	50	20
Wheezing	20	9
Rash (yes/no)	20	—
Hand–foot syndrome	18	4

Note

All toxicities refer to severity unless otherwise noted.

“Any” refers to a score of 1 or more; “Moderate to very severe” refers to a score of 2 or more.

Severity: 0=none, 1=mild, 2=moderate, 3=severe, 4=very severe.

Frequency: 0=never, 1=rarely, 2=occasionally, 3=frequently, 4=almost constantly.

Yes/no: 0=no, 1=yes.

## 4 DISCUSSION

To our knowledge, this study is the first to describe trajectories of PROs among adult recipients of axi‐cel. Data suggest that axi‐cel is associated with favorable PROs. Notably, axi‐cel recipients reported increases in anxiety from baseline to day 90. Anxiety may be especially heightened in axi‐cel recipients because they have active disease prior to treatment and progression is expected to occur in most cases within 90 days. Further, many participants completed the QOL measure before they knew their day 90 scan results.

Changes in QOL were not associated with disease response, CRS, or neurotoxicity. These results are consistent with studies of HCT recipients reporting no association between QOL and length of hospital stay, a proxy for posttransplant complications.^32^, ^33^ Nevertheless, caution is warranted when interpreting these findings since the analyses were exploratory and likely underpowered. Additional studies with larger samples are needed to confirm these results.

A secondary goal of this study was to evaluate patient‐reported toxicity of axi‐cel to provide a more complete picture of patient functioning during the acute treatment period. Patient‐reported toxicities peaked at 14 days after CAR T‐cell infusion, with the majority of toxicities improving by day 90. The exception to this pattern was muscle aches, which persisted at day 90. The overall trajectory of symptoms is similar to reported symptom burden in allogeneic HCT with an acute peak during the peri‐treatment period, then, a return to baseline of most symptoms by day 100.^34^ In comparison to adverse events reported on the registration trial for axi‐cel,^1^, ^2^ patient‐reported toxicities tended to be more common and severe in this study. This finding supports measurement of PROs to complement physician‐reported toxicities in this novel therapy.

The current study is characterized by several strengths, including a novel and clinically important question, use of well‐validated and CMS‐recommended PRO measures, and prospective data collection. Limitations of this study should also be noted, however. In total, nearly one‐third of axi‐cel recipients who provided baseline data did not provide data at 90 days due to putative morbidity and mortality (i.e., reasons other than not due for the 90 assessment yet). Our attrition rate compares favorably to observational studies of HCT,^35^ in which attrition due to morbidity and mortality is not uncommon. A previous study evaluating the bias of attrition on QOL in HCT recipients has shown that attrition contributes to slight overestimation of QOL that is stable across time.^32^ Another limitation of the study is that patients completed assessments while they were inpatient, thus, they may have had limited insight into some of their symptoms. However, PROs capture patients’ own perspectives of their abilities and symptoms, and they are taken at their word in terms of what they are experiencing. Finally, knowledge of adverse events of axi‐cel was limited to a single published study^2^ when we selected PRO‐CTCAE items. Thus, there may be additional patient‐reported toxicities that we did not capture, such as swelling, heart palpitations, and dizziness. Future studies should revisit the question of whether additional patient‐reported toxicities should be assessed.

In summary, the current study suggests improvement or stability in the majority of QOL domains after axi‐cel with transient worsening of most patient‐reported toxicities. In the rapidly growing field of adaptive cellular therapy, CAR T‐cell therapy is being studied earlier in the treatment course and is being expanded to other tumor types. As such, it is important to understand PROs to allow for comprehensive discussions with patients regarding the risks and benefits, including tolerability, of this therapy. Continued evaluation of PROs with longer follow‐up is needed to understand the survivorship needs of this unique population of patients. Additional studies are needed to evaluate supportive care interventions to further improve QOL in patients treated with CAR T‐cell therapy. Potential interventions include early physical therapy for those with fatigue or poor physical function, as well as early evaluation and treatment with a mental health provider for those with anxiety or depression.

## CONFLICTS OF INTEREST

Jain: consultant—Kite/Gilead, Novartis. Bachmeier: advisor—Kite/Gilead. Shah: advisor/consultant—Kite/Gilead, Novartis, Celgene/Juno, Spectrum/Acrotech, AstraZeneca, Pharmacyclics; grant/IIT—Jazz, Kite, Incyte. Locke: scientific advisor—Kite, Novartis, BMS/Celgene, Allogene, Amgen, Calibr, Wugen, GammaDelta Therapeutics; consultant—Cellular Biomedicine Group Inc.; research funding—Kite; intellectual property—patents in the field of cellular therapy held by employer in Locke's name. Jim: consultant—RedHill Biopharma, Janssen Scientific Affairs, and Merck.

## AUTHOR CONTRIBUTIONS

Conceptualization: HSLJ and FLL; funding acquisition: HSLJ and FLL; methodology: HSLJ and FLL; formal analysis: AIH; investigation: AC and NIA; data curation: AIH, RVJ, MDJ, KAH, BWJ, and AB; visualization: AIH and RVJ; resources: MDJ, MBJ, CAB, JCC, FK, GSK, AL, HDL, TN, JPI, BDS, and MA; supervision: MDJ, HSLJ, and FLL; project administration: YR; writing—original draft preparation: all authors; writing—review and editing—all authors.

## Supporting information

Fig S1

Table S2

Table S3

## Data Availability

The data that support the findings of this study are available from the corresponding author upon reasonable request.
