# Supplementary material for: Acute patient‐reported outcomes in B‐cell malignancies treated with axicabtagene ciloleucel
Source: Cancer Med. 2021 Feb 28;10(6):1936–43. doi: 10.1002/cam4.3664 (PMC7957158; doi:10.1002/cam4.3664)
Supplement: Supplementary file 1 — Fig S1 [file CAM4-10-1936-s001.docx]

**Supplemental Figure 1.** CONSORT diagram

## Consented (n = 163)

Did not provide baseline data (n=61)

- Withdrew (n=3)
- Ineligible after consent (n=7)
- Screening failure (n=1)
- Dropped by research team (n=2)
- Received different I/O product (n=38)
- Received diagnosis of ALL (n=8)
- Did not return baseline questionnaire (n=2)

## Baseline assessment (n=102)

Did not provide 14-day follow-up data (n=15)

- Withdrew (n=1)
- Died (n=5)
- Did not return 14-day questionnaire (n=9)

Did not provide 30-day follow-up data (n=10)

- Did not return 30-day questionnaire (n=8)
- Died (n=1)
- Dropped by research team (n=1)

Provided 30-day data but not 14-day data (n=9)

Provided 30-day data but not 14-day data (n=?)

## 14-day follow-up assessment (n=87)

## 30-day follow-up assessment (n=86)

Did not provide 60-day follow-up data (n=7)

- Died (n=3)
- Did not return 60-day questionnaire (n=2)
- Lost to follow-up (n=1)
- Withdrew (n=1)

Provided 60-day data but not 30-day data (n=8)

## 60-day follow-up assessment (n=87)

Did not provide 90-day follow-up data (n=17)

- Withdrew (n=5)
- Did not return 90-day questionnaire (n=12)

Provided 90-day data but not 60-day data (n=2)

## 90-day follow-up assessment (n=72)
