# Supplementary material for: Acute patient‐reported outcomes in B‐cell malignancies treated with axicabtagene ciloleucel
Source: Cancer Med. 2021 Feb 28;10(6):1936–43. doi: 10.1002/cam4.3664 (PMC7957158; doi:10.1002/cam4.3664)
Supplement: Supplementary file 2 — Table S2 [file CAM4-10-1936-s003.docx]

| **Supplemental Table 2**. Self-reported toxicity means and standard deviations in patients receiving axi-cel therapy | | | | | |
| --- | --- | --- | --- | --- | --- |
| **Symptom** | **Baseline** | **14 Days** | **30 Days** | **60 Days** | **90 Days** |
| Fatigue | 1.29 (1.03) | 2.07 (1.1) | 1.69 (1.07) | 1.13 (.9) | 1.08 (.91) |
| Decreased appetite | .85 (1) | 1.98 (1.18) | 1.09 (1.12) | .37 (.73) | .29 (.72) |
| Dry mouth | .9 (1.05) | 1.7 (1.16) | .76 (.93) | .46 (.83) | .44 (.89) |
| Diarrhea (frequency) | .67 (1.11) | 1.13 (1.24) | .55 (.84) | .49 (.91) | .44 (.92) |
| Problems with concentration | .46 (.71) | 1.05 (1.09) | .73 (.8) | .6 (.75) | .42 (.67) |
| Insomnia | 1.07 (1.1) | 1.01 (1.16) | .95 (1) | .82 (.97) | .8 (1.06) |
| Constipation | .72 (.98) | .93 (1.22) | .66 (.93) | .34 (.7) | .25 (.69) |
| Headache | .36 (.65) | .91 (1.11) | .64 (.85) | .45 (.73) | .49 (.75) |
| Problems with memory | .44 (.66) | .91 (1.01) | .55 (.73) | .56 (.79) | .52 (.71) |
| Aching muscles | .58 (.82) | .91 (.9) | .76 (.95) | .83 (.9) | .83 (.96) |
| Sad or unhappy | .69 (.77) | .87 (.97) | .64 (.72) | .51 (.71) | .55 (.75) |
| Nausea | .45 (.79) | .83 (1.05) | .49 (.89) | .29 (.68) | .24 (.57) |
| Aching joints | .45 (.79) | .75 (1.03) | .57 (.86) | .68 (.93) | .82 (.88) |
| Abdominal pain | .59 (.94) | .64 (1.04) | .51 (.9) | .4 (.75) | .38 (.78) |
| Shortness of breath | .6 (.93) | .62 (.84) | .63 (.9) | .55 (.9) | .38 (.59) |
| Cough | .35 (.74) | .52 (.83) | .62 (.94) | .31 (.69) | .39 (.72) |
| Hair loss (yes/no) | .07 (.26) | .44 (.5) | .34 (.48) | .13 (.34) | .11 (.32) |
| Itchy skin | .27 (.66) | .41 (.74) | .24 (.59) | .22 (.58) | .22 (.51) |
| Hand-foot syndrome | .03 (.17) | .14 (.38) | .08 (.41) | .06 (.28) | .01 (.12) |
| Rash (yes/no) | .05 (.22) | .14 (.35) | .07 (.26) | .03 (.18) | .03 (.17) |
| Wheezing | .14 (.51) | .1 (.37) | .07 (.26) | .06 (.35) | .08 (.33) |
| Note: All toxicities refer to severity unless otherwise noted. | | | |  |  |
| Severity: 0=none, 1=mild, 2=moderate, 3=severe, 4=very severe | | | |  |  |
| Frequency: 0=never, 1=rarely, 2=occasionally, 3=frequently, 4=almost constantly | | | | | |
| Yes/no: 0=no, 1=yes |  |  |  |  |  |
