# Supplementary material for: Acute patient‐reported outcomes in B‐cell malignancies treated with axicabtagene ciloleucel
Source: Cancer Med. 2021 Feb 28;10(6):1936–43. doi: 10.1002/cam4.3664 (PMC7957158; doi:10.1002/cam4.3664)
Supplement: Supplementary file 3 — Table S3 [file CAM4-10-1936-s002.docx]

| **Supplemental Table 3**. Patient-reported toxicity changes in time: | | |
| --- | --- | --- |
| Mixed model parameter estimates | |  |
| **Variable** | **Time** | **Time*Time** |
| Dry mouth | 0.13 | -0.08*** |
| Decreased appetite | 0.43*** | -0.17*** |
| Nausea | 0.13 | -0.06** |
| Constipation | 0.04 | -0.05 |
| Diarrhea (frequency) | 0.06 | -0.04 |
| Abdominal pain | -0.02 | -0.01 |
| Shortness of breath | 0.1 | -0.03 |
| Cough | 0.17* | -0.04* |
| Wheezing | -0.05 | 0.01 |
| Rash (yes/no) | 0.03 | -0.01 |
| Hair loss (yes/no) | 0.25*** | -0.07*** |
| Itchy skin | 0.01 | -0.01 |
| Hand-foot syndrome | 0.07* | -0.02* |
| Problems with concentration | 0.37*** | -0.11*** |
| Problems with memory | 0.19** | -0.05** |
| Headache | 0.27** | -0.07*** |
| Aching muscles | 0.19* | -0.03 |
| Aching joints | 0.09 | -0.01 |
| Insomnia | -0.09 | 0 |
| Fatigue | 0.44*** | -0.14*** |
| Sad or unhappy | 0.01 | -0.02 |
| **p*<0.05, ***p*<0.01, ****p*<0.001 | |  |
